# Dynamic Changes in the Splenic Transcriptome of Chickens during the Early Infection and Progress of Marek’s Disease

**DOI:** 10.1038/s41598-017-11304-y

**Published:** 2017-09-14

**Authors:** Lu Dang, Man Teng, Hua-Wei Li, Hui-Zhen Li, Sheng-Ming Ma, Pu Zhao, Xiu-Jie Li, Rui-Guang Deng, Gai-Ping Zhang, Jun Luo

**Affiliations:** 1 College of Veterinary Medicine, Northwest A&F University, Yangling, 712100 People’s Republic of China; 2 Key Laboratory of Animal Immunology of the Ministry of Agriculture, Henan Provincial Key Laboratory of Animal Immunology, Henan Academy of Agricultural Sciences, Zhengzhou, 450002 People’s Republic of China; 3 College of Animal Science and Veterinary Medicine, Henan Agricultural University, Zhengzhou, 450002 People’s Republic of China; 4 College of Animal Science and Technology, Henan University of Science and Technology, Luoyang, 471003 People’s Republic of China; 5 Jiangsu Co-innovation Center for Prevention and Control of Important Animal Infectious Diseases and Zoonoses, Yangzhou, 225009 People’s Republic of China

## Abstract

*Gallid alphaherpesvirus 2* (GaHV2) is an oncogenic avian *herpesvirus* inducing Marek’s disease (MD) and rapid-onset T-cell lymphomas. To reveal molecular events in MD pathogenesis and tumorigenesis, the dynamic splenic transcriptome of GaHV2-infected chickens during early infection and pathogenic phases has been determined utilizing RNA-seq. Based on the significant differentially expressed genes (DEGs), analysis of gene ontology, KEGG pathway and protein-protein interaction network has demonstrated that the molecular events happening during GaHV2 infection are highly relevant to the disease course. In the ‘Cornell Model’ description of MD, innate immune responses and inflammatory responses were established at early cytolytic phase but persisted until lymphoma formation. Humoral immunity in contrast began to play a role firstly in the intestinal system and started at late cytolytic phase. Neurological damage caused by GaHV2 is first seen in early cytolytic phase and is then sustained throughout the following phases over a long time period. During the proliferative phase many pathways associated with transcription and/or translation were significantly enriched, reflecting the cell transformation and lymphoma formation. Our work provides an overall view of host responses to GaHV2 infection and offers a meaningful basis for further studies of MD biology.

## Introduction

Marek’s disease (MD) is a major infectious disease affecting poultry health worldwide and is responsible for an approximate annual global economic losses of $2 billion^1^. MD is characterized in the early stages by transient neurological signs and immunosuppression, followed by lymphoma formation in susceptible breeds in various visceral organs^2, 3^. The causative agent, *Gallid alphaherpesvirus 2* (GaHV2), commonly known as Marek’s disease virus type 1 (MDV-1), is an oncogenic avian herpesvirus belonging to the subfamily *Alphaherpesvirinae* (https://talk.ictvonline.org/taxonomy/). GaHV2 has a long and complex pathogenic life cycle, which has been well established as the ‘Cornell Model’^4^ and includes four phases: (a) the early cytolytic phase [2–7 days post-infection (dpi)], (b) the latent phase (7–10 dpi onwards), (c) the late-cytolytic and immunosuppressive phase (18 dpi onwards) and (d) the proliferative phase (28 dpi onwards)^5^. However little is known of the molecular mechanisms underlying the course of disease.

The available chicken genome database^6^ and microarrays^7^ have become useful tools to study the gene and protein expression profiles of GaHV2-host interaction and for further revealing the molecular mechanisms involved in the pathogenic and tumorigenic responses to GaHV2 infection. Previously microarray analysis has been performed *in vitro* of the interactions between host cells and GaHV2, such as in the primary chicken embryo fibroblasts (CEFs) and the transformed cell line DF-1^8, 9^. This technology has also been used to investigate gene expression changes *in vivo* ^10^. Recently, several studies have been independently performed on the liver, thymus, spleen, or bursa of Fabricius of GaHV2-infected chickens^11–15^. However, a dynamic genome wide transcriptome of host responses to GaHV2 infection during the course of disease remains unclear.

Next generation sequencing (NGS) is a high-throughput technology with great power for genome-wide analysis, which has been successfully applied in various aspects of the life sciences, covering DNA^16^, RNA^17^, and epigenetics^18^. Currently, the advanced and alternative high-throughput platforms for RNA sequencing (RNA-seq), such as Roche’s 454 GS FLX, Illumina/Solexa HiSeq2000 and Applied Biosystems’ SOLiD^19^, can obtain greater sequence coverage and contribute a lot to facilitate the assembly of transcripts and to identify rare transcripts. Herein, utilizing RNA-seq, we have performed a comprehensive analysis of the transcriptomes of GaHV2-infected chickens during the virus life cycle and the development of MD lymphomas. Based on the high-throughput technology, the dynamic and differential gene expression profiles obtained from GaHV2-infected chicken spleens will provide an important basis and more valuable information for future studies on the molecular mechanisms of MD pathogenesis and tumorigenesis.

## Results

### Overview of RNA-seq and data processing

To reveal the dynamics of transcription in the spleens of GaHV2-infected chickens, a series of cDNA libraries including six from the GX0101-infected birds viz- CH-3dpi, CH-7dpi, CH-14dpi, CH-21dpi, CH-30dpi, and CH-60dpi together with six of the mock-infected birds viz- CH-3d, CH-7d, CH-14d, CH-21d, CH-30d, and CH-60d respectively, were simultaneously constructed for sequencing. As shown in Table 1, the RNA-seq generated 14,921,302 to 24,827,231 raw reads for GX0101-infected groups at the time points from 3 to 60 dpi. As for the corresponding mock groups, 15,369,671 to 19,565,208 raw reads were obtained at each time point. After filtering (Table S1), the clean reads accounted for more than 97.5% of the raw reads were used for the subsequent analyses (Fig. S1). The quality of the clean sequences was good with more than 96.8% of which possessed a Phred quality score of Q30 level (error probability of 0.1%). Aligning all cleaned reads to the chicken reference genome, totals of 14,363,551, 15,845,242, 13,116,631, 13,393,436, 22,112,729 and 14,936,536 mapped reads were obtained from the GX0101-infected cDNA libraries (Table 1). Similarly, there were 17,540,562, 16,603,424, 15,323,583, 15,940,173, 22,112,730 and 13,685,458 mapped reads harvested in the six mock control libraries, accounting for at least 89.7% of the clean reads. A small proportion of the clean reads (about 2%) were mapped to multiple locations in the genome. The twelve cDNA libraries from GX0101-infected birds or mock controls shared similar proportions of gene distributions (Fig. S2), of which there were approximately 57–64%, 11–17%, and 24–27% clean reads mapped to the exon, intron and intergenic regions respectively.

**Table 1:** Summary of the RNA-seq data of cDNA libraries constructed from GX0101-infected or mock-infected chickens.

Group	Category	cDNA libraries constructed at different days post infection (dpi)											
		CH-3 dpi/CH-3d		CH-7 dpi/CH-7d		CH-14 dpi/CH-14d		CH-21 dpi/CH-21d		CH-30 dpi/CH-30d		CH-60 dpi/CH-60d	
		Counts	%	Counts	%	Counts	%	Counts	%	Counts	%	Counts	%
GX0101-infected	Raw reads	16,089,905	—	17,921,467	—	14,921,302	—	15,335,675	—	24,827,231	—	16,764,369	—
	Clean reads	15,720,080	97.71	17,528,208	97.83	14,587,807	97.86	14,995,303	97.83	24,290,700	97.87	16,412,919	97.92
	Q30 bases	—	96.83	—	96.98	—	96.85	—	96.97	—	97.09	—	96.91
	Mapped reads	14,363,551	91.37	15,845,242	90.40	13,116,631	89.92	13,393,436	89.32	22,112,729	91.03	14,936,536	91.00
	Multi mapped reads	295,645	2.06	360,752	2.28	291,445	2.22	335,886	2.51	546,379	2.47	297,506	1.99
	Unmapped reads	1,356,529	8.63	1,682,966	9.60	1,471,176	10.10	1,601,867	10.70	2,177,971	8.97	1,476,383	9.00
Mock-infected	Raw reads	19,565,208	—	18,636,075	—	17,509,762	—	17,994,555	—	18,753,333	—	15,369,671	—
	Clean reads	19,125,928	97.85	18,214,103	97.7	17,076,889	97.58	17,584,629	97.79	18,334,284	97.82	15,037,960	97.88
	Q30 bases	—	96.82	—	96.87	—	96.71	—	96.86	—	96.87	—	96.93
	Mapped Reads	17,540,562	91.71	16,603,424	91.24	15,323,583	89.75	15,940,173	90.72	22,112,730	91.03	13,685,458	91.01
	Multi mapped reads	391,013	2.23	364,817	2.20	372,080	2.43	348,472	2.19	384,323	2.32	311,799	2.28
	Unmapped reads	1,585,334	8.29	1,610,659	8.84	1,753,317	10.32	1,644,512	9.35	1,769,820	9.65	1,352,514	8.99

For each time point, the cDNA libraries constructed for GX0101-infected birds or mock controls were named as CH-#dpi and CH-#d, respectively. ‘−’ Not applicable.

### DEGs in GaHV2 infection obtained from RNA-seq

The DEGs in GX0101-infected birds compared to the mock controls were further analyzed. The expression levels of chicken genes were estimated by RPKM values and are listed in Table S2. The criteria set for fold change ≥ 2 and FDR < 0.05 were used as the threshold to identify significant differences in gene expression. As shown in Table 2, totals of 1,567, 1,342, 2,503, 3,517, 3,810 and 1,351 genes, listed in Table S3, were significantly differentially expressed at 3, 7, 14, 21, 30 and 60 dpi, respectively. Among these DEGs, 736, 812, 1,507, 2,250, 2,390 and 571 genes were observed to be up-regulated during viral infection at 3 to 60 dpi, along with the simultaneous down-regulation of 831, 530, 996, 1,267, 1,420 and 780 genes. The numbers of DEGs up-regulated and down-regulated present a similar trend to increase from 7 to 21 dpi, reaching a peak at 30 dpi and then decrease by 60 dpi.

**Table 2:** The number of up- or down-regulated DEGs based on pair-wise comparison with mock control (fold change ≥2; FDR <0.05).

Groups	Up-regulated	Down-regulated	Total
CH-3dpi Vs CH-3d	736	831	1,567
CH-7dpi Vs CH-7d	812	530	1,342
CH-14dpi Vs CH-14d	1,507	996	2,503
CH-21dpi Vs CH-21d	2,250	1,267	3,517
CH-30dpi Vs CH-30d	2,390	1,420	3,810
CH-60dpi Vs CH-60d	571	780	1,351

### Validation of the DEGs during the course of MD

**Figure 1:**
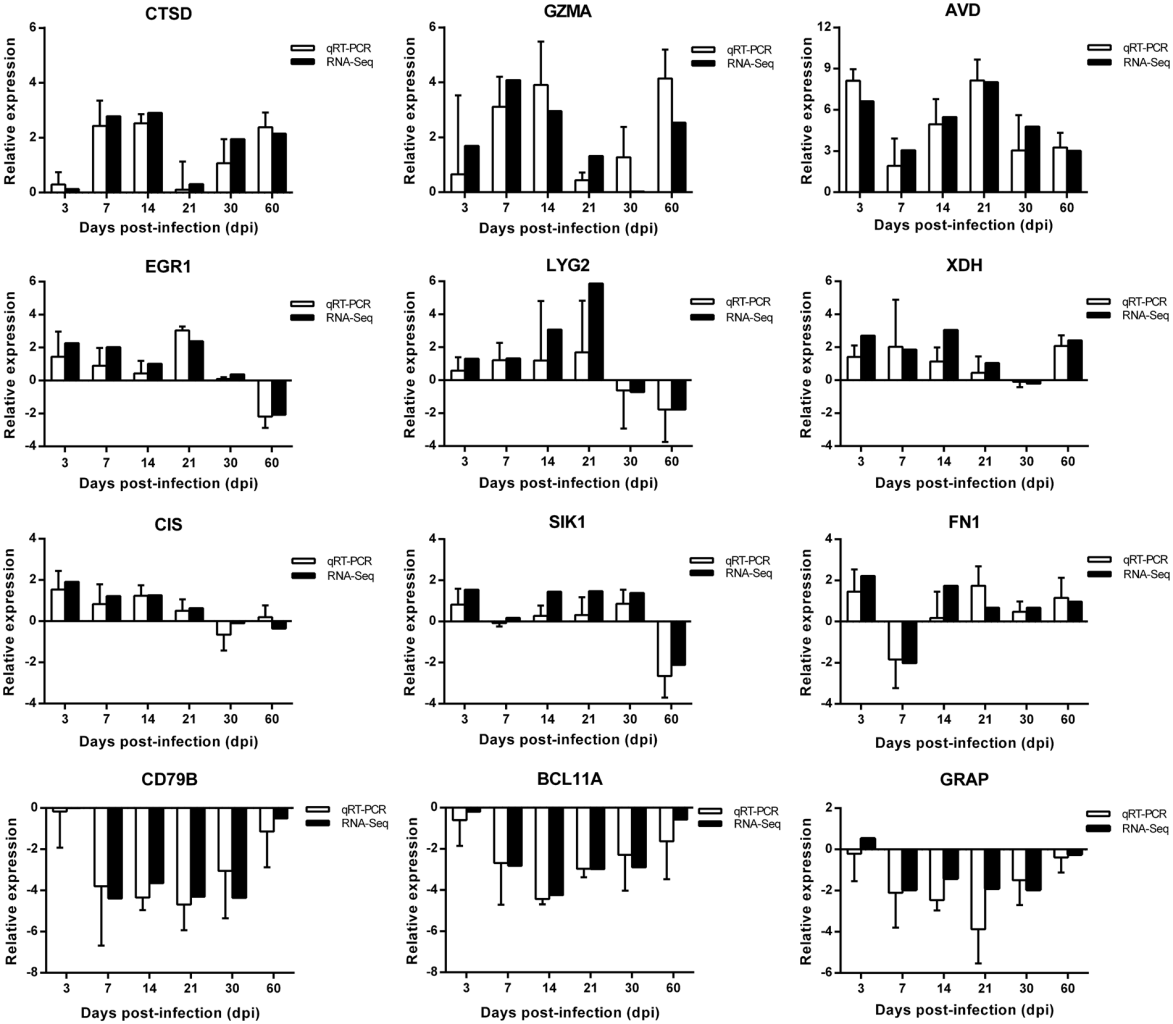
Validation of differentially expressed genes by qRT-PCR. The results of qRT-PCR were normalized to host *GAPDH* gene for the same samples. Relative expression levels of 12 chicken genes determined by qRT-PCR and RNA-seq are shown by white or black bars, respectively. Error bar indicates standard error (SE) of the mean.

The qRT-PCR was performed to validate the DEGs obtained from RNA-seq. A total of 12 target genes (Table S4) representing a good coverage of both up- or down-regulated genes in GaHV2 infection were randomly selected for qRT-PCR confirmation. The house-keeping gene *GAPDH* was simultaneously validated as an appropriate endogenous reference gene. Even though some of the genes selected for validation did not show significant changes in expression at all of the time points, all selected genes were put through the validation procedure during the whole course of disease. As demonstrated in Fig. 1, for all of the 12 host genes, the qRT-PCR experiments exhibited consistent results to those of RNA-seq. Five of chicken immune/stress response genes were confirmed to be significantly differentially expressed, including four up-regulated genes LYG2, C1S, GZMA, CTSD and one down-regulated gene CD79B. Besides, five genes related to cell differentiation or tumorigenesis were similarly confirmed, containing three up-regulated genes EGR1, SIK1, FN1 and two down-regulated genes BCL11A and GRAP. Thus, the RNA-seq data was well supported by the qRT-PCR confirmation allowing it to provide the basis for the following analysis.

### K-means clustering of DEGs

**Figure 2:**
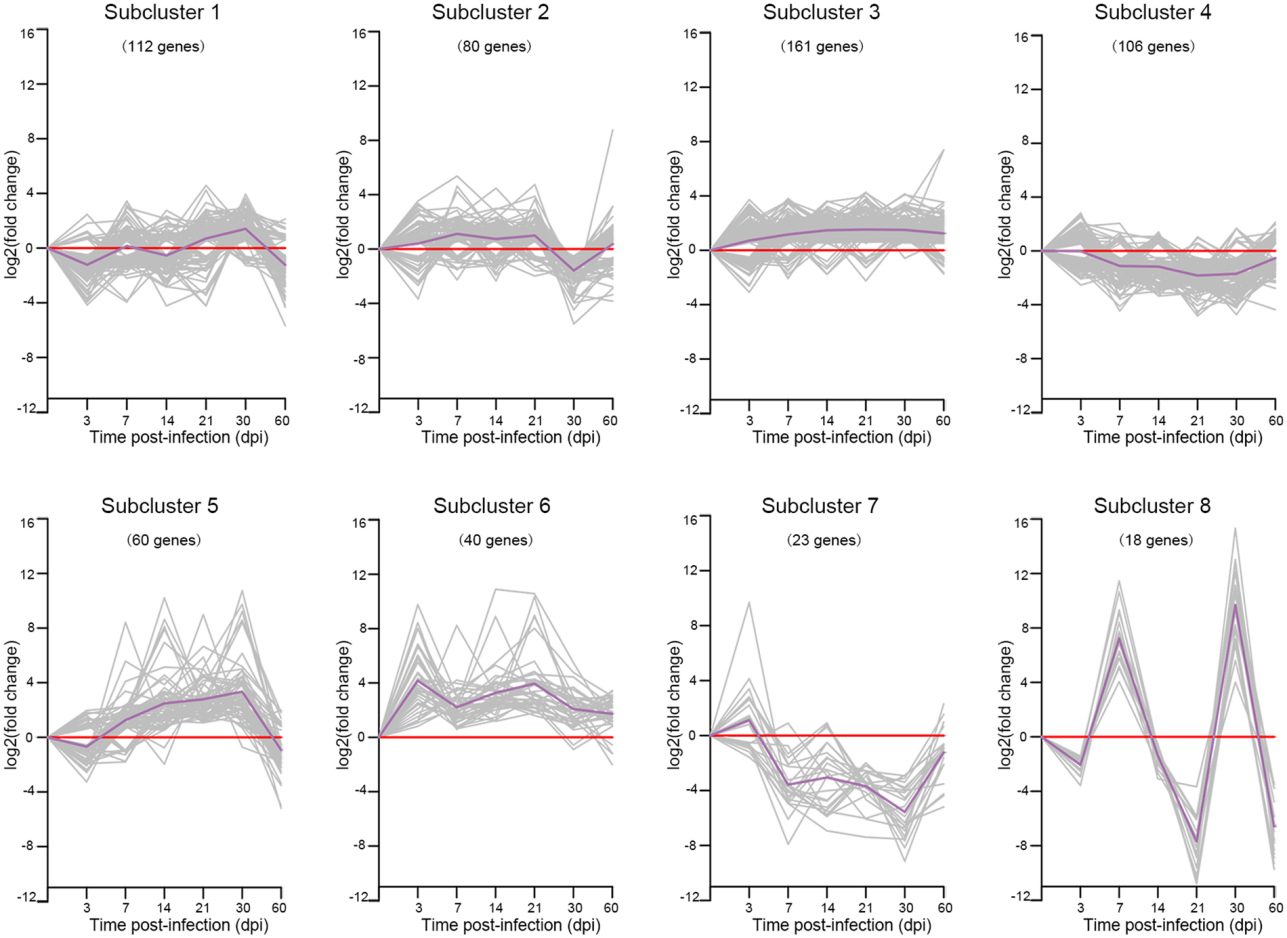
K-means clustering of DEGs. The DEGs based on |log2 (fold change) | ≥ 0.5 were statistically grouped into 8 subclusters. The trends of distinct significant expression subclusters were analysed.

To further investigate the biological characteristics of the DEGs, K-mean cluster analysis was performed. The DEGs with |log_2_(fold change) | ≥ 0.5 were statistically grouped into eight subclusters based on the expression profile similarity at different time points post GaHV2 infection. As shown in Fig. 2, the subclusters 1, 2, 3 and 4 contain 112, 80, 161 or 106 genes, respectively. The gene expression patterns of these genes showed slight fluctuant changes during the processes of GaHV2 infection. Genes in these four clusters were predominantly enriched related with regulation of growth and cell fate commitment based on GO analysis. GO terms enriched in subclusters 5 and 6 included defense response to virus, regulation of apoptotic process, cytokine activity and extracellular space, suggesting the importance of the genes that response to signal transmission after GaHV2 infection. Within contrast to subclusters 5 and 6, genes in subcluster 7 showed an opposite trend and were down-regulated in GaHV2 infection. Particularly, the subcluster 8 contains 18 genes, including HINTW, WPKCI, RPL17L, etc. These genes represented a waved expression pattern - increased firstly, a sharp decrease at 21 dpi, followed by a huge up-regulation at 30 dpi, and ended in a down-regulation at 60 dpi.

### GO and KEGG enrichment analysis of DEGs

**Figure 3:**
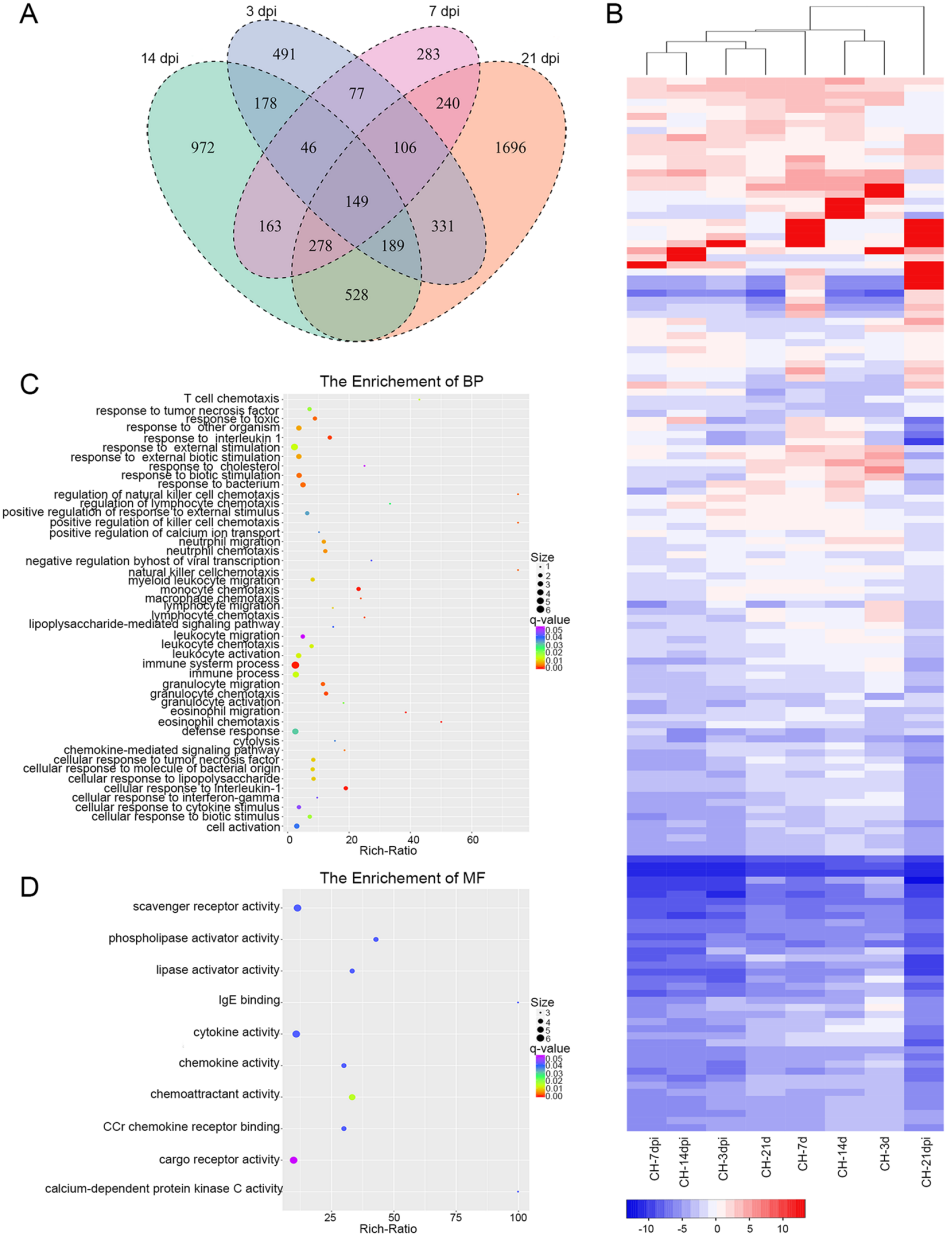
Analysis of DEGs among 3, 7, 14 and 21 dpi. (**A**) Venn diagram showing the overlap between DEGs. (**B**) A hierarchical clustering of overlapped DEGs was obtained using RNA-seq data that was derived from the four time pints based on log_2_ RPKM values. The blue bands indicate low gene expression levels, and the red bands indicate high gene expression levels. (**C**) The enriched biological process terms of overlapped genes. (**D**) The enriched molecular function terms of overlapped genes.

**Figure 4:**
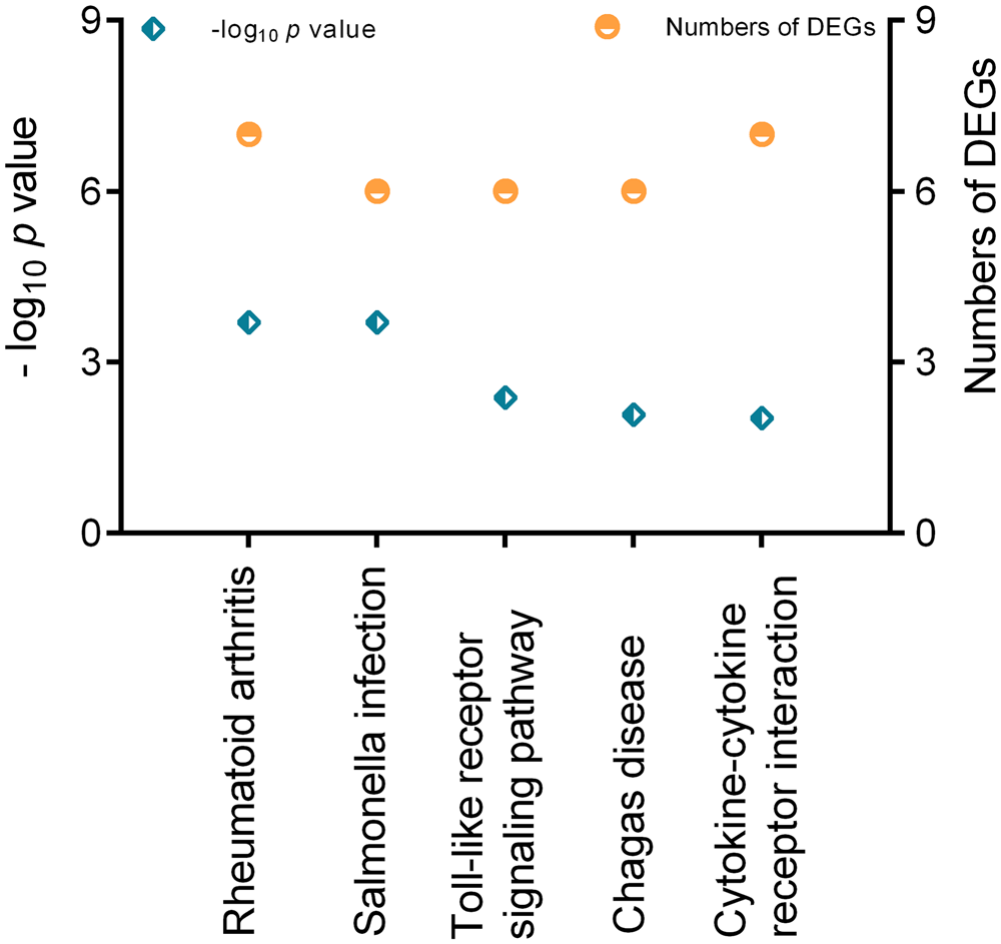
Enriched KEGG pathways for overlapped DEGs among 3, 7, 14 and 21 dpi. The left y-axis shows the −log_10_ (*p*-value) and the right shows the numbers of involved DEGs.

For further analyzing DEGs among different time points post infection, we performed two groups of comparative analysis. As shown in Fig. 3A, there were 149 genes shared among 3, 7, 14 and 21 dpi. The hierarchical clustering of these DEGs based on the log_10_(RPKM + 1) values for each of the different groups are demonstrated in Fig. 3B. The GO enrichment analysis of overlapped genes showed that subclasses like eosinophil chemotaxis, cellular response to interleukin-1, immune system process, eosinophil migration, monocyte chemotaxis, response to interleukin-1, lymphocyte chemotaxis, natural killer cell chemotaxis and regulation of natural killer cell chemotaxis, etc, were enriched within the category of biological process (Fig. 3C). Ten subclasses of molecular function stood outstanding (Fig. 3D) while only one subclass of cellular component, extracellular space, was enriched. The KEGG analysis showed five pathways, Rheumatoid arthritis, Salmonella infection, Toll-like receptor signaling pathway, Chagas disease and Cytokine-cytokine receptor interaction, were significantly enriched in 3–21 days post GaHV2 infection (Fig. 4).

**Figure 5:**
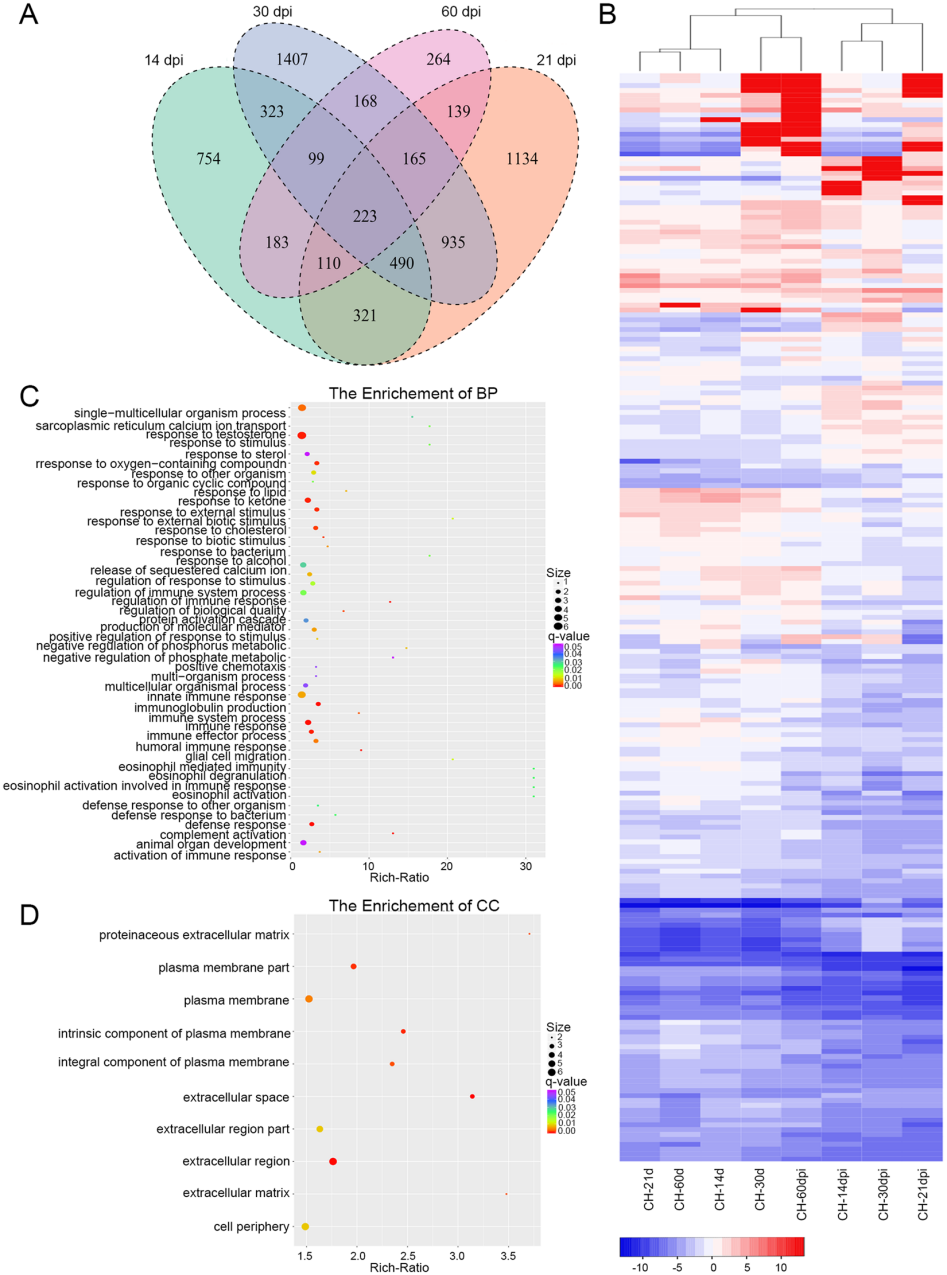
Analysis of DEGs among 14, 21, 30 and 60 dpi. (**A**) Venn diagram showing the overlap between DEGs. (**B**) A hierarchical clustering of overlapped DEGs was obtained using RNA-seq data that was derived from the four time pints based on log_2_ RPKM values. The blue bands indicate low gene expression levels, and the red bands indicate high gene expression levels. (**C**) The enriched biological process terms of overlapped genes. (**D**) The enriched cellular component terms of overlapped genes.

For the comparative analysis of 14, 21, 30 and 60 dpi, a total of 223 overlapped DEGs were observed (Fig. 5A) and the heatmap of these genes was shown as Fig. 5B. Forty-nine subterms of biological process were annotated, such as protein activation cascade, defense response, complement activation, humoral immune response and negative regulation of phosphorus metabolic process (Fig. 5C). For the category of cellular component, 10 subterms were highlighted (Fig. 5D), but only one term of antigen binding was enriched for molecular function. No significant enriched pathways were observed in 14–60 days post GaHV2 infection.

The comparative analysis covering 3 time series (7, 14 & 21 or 21, 30 & 60) and 2 time series (14 & 21, 21 & 30, or 30 & 60) were also performed but no significant pathway was enriched (data not shown). The DEGs at each time point post GaHV2 infection were functionally annotated. As show in Table 3, at both 3 and 7 dpi the DEGs were significantly enriched for the same two pathways of Cytokine-cytokine receptor interaction and the Hematopoietic cell lineage. In addition, the Complement and coagulation cascades and Rheumatoid arthritis were significantly enriched at 3 dpi and 7 dpi, respectively. The DEGs at 14 dpi were significantly enriched into eight KEGG pathways. Except for ‘Cytokine-cytokine receptor interaction’ the rest of the significantly enriched pathways correlated with immune responses include the ‘Intestinal immune network for IgA production’ and ‘JAK-STAT signaling pathway’, in the latter of which several genes such as IL6, IFN, JAK, PIAS, SOCS and BclXL were up-regulated while IL2/3 and CycD were down-regulated. More significant pathways were represented at 21 dpi (Table 3). Elements from the gene transcription, mRNA processing and protein translation related pathways, such as the ‘Ribosome biogenesis in eukaryotes’, ‘Spliceosome’ and ‘Ribosome’, made an appearance. A pathway related to signaling molecules and interaction, namely ‘Cell adhesion molecules (CAMs)’, also was noted. Additionally, the pathway ‘Epstein-Barr virus infection’ was simultaneously enriched, in which several genes including BCL2, CD39, HSP70, HDAC45, IL10, IL10R and vimentin were up-regulated, together with some down-regulated genes such as CD38, NUP214, RBP-Jκ, CycA, TAK1 and TBK1. At this phase, as shown in Table 3, several metabolic related pathways were also significantly enriched. A total of six significant pathways were enriched at 30 dpi, five of which had been observed before this time point and a new significant pathway, ‘mRNA surveillance pathway’, was first enriched at this time. The pathway of ‘Neuroactive ligand-receptor interaction’ was persistently enriched from 14 to 60 dpi (Table 3).

**Table 3:** Significant KEGG pathways analyzed based on the differentially expressed genes (DEGs) at each time point.

Time point	Entry	KEGG description	*p*-value
3 dpi	map04060	Cytokine-cytokine receptor interaction	5.42E-07
	map04640	Hematopoietic cell lineage	1.72E-02
	map04610	Complement and coagulation cascades	4.71E-02
7 dpi	map04060	Cytokine-cytokine receptor interaction	1.23E-02
	map04640	Hematopoietic cell lineage	1.99E-02
	map05323	Rheumatoid arthritis	1.23E-02
14 dpi	map04060	Cytokine-cytokine receptor interaction	7.45E-08
	map04630	Jak-STAT signaling pathway	4.26E-02
	map04672	Intestinal immune network for IgA production	3.51E-02
	map05323	Rheumatoid arthritis	4.01E-02
	map04080	Neuroactive ligand-receptor interaction	2.74E-04
	map05320	Autoimmune thyroid disease	1.10E-02
	map03013	RNA transport	3.51E-02
	map04976	Bile secretion	4.26E-02
21 dpi	map04060	Cytokine-cytokine receptor interaction	1.24E-02
	map04610	Complement and coagulation cascades	1.24E-02
	map04672	Intestinal immune network for IgA production	1.24E-02
	map04080	Neuroactive ligand-receptor interaction	1.24E-02
	map03008	Ribosome biogenesis in eukaryotes	1.24E-02
	map03040	Spliceosome	5.69E-04
	map03010	Ribosome	3.74E-03
	map04514	Cell adhesion molecules (CAMs)	4.09E-02
	map05169	Epstein-Barr virus infection	4.09E-02
	map00250	Alanine, aspartate and glutamate metabolism	1.24E-02
	map00910	Nitrogen metabolism	1.35E-02
	map04972	Pancreatic secretion	4.51E-02
	map04975	Fat digestion and absorption	1.42E-02
30 dpi	map04060	Cytokine-cytokine receptor interaction	6.22E-05
	map04080	Neuroactive ligand-receptor interaction	6.22E-05
	map03008	Ribosome biogenesis in eukaryotes	3.78E-03
	map03040	Spliceosome	6.22E-05
	map03013	RNA transport	6.72E-03
	map03015	mRNA surveillance pathway	6.72E-03
60 dpi	map04080	Neuroactive ligand-receptor interaction	3.45E-02

### Protein-protein interaction networks in GaHV2 infection

**Figure 6:**
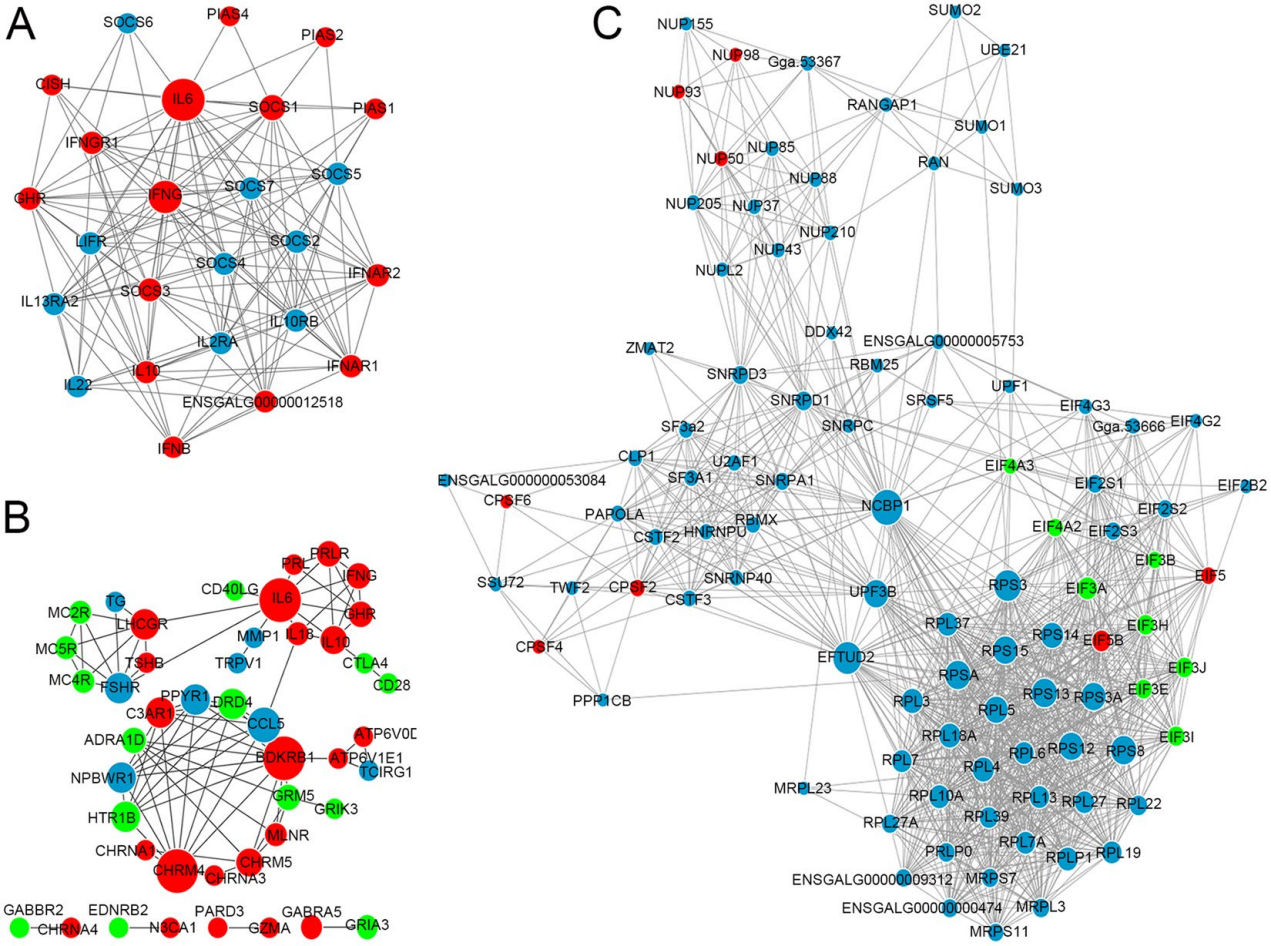
The PPI networks of negative regulators of JAK/STAT signaling (**A**), neurological damage (**B**) and gene transcription and translation (**C**). The red, green and blue circles indicate the up-, down-regulated or unchanged genes, respectively. The size of nodes is positively correlated to their degree of connectivity. Edges are shown by grey lines and indicate direct interactions.

The significant DEGs were submitted to the STRING database for analyzing the protein-protein interaction (PPI) pairs, which were further loaded into Cytoscape software to construct PPI networks. As demonstrated in Fig. 6A, a PPI network related to the negative regulators of JAK/STAT signaling containing 26 nodes and 144 edges (line connections between nodes) was obtained. In this network, IL6, IFN-γ, suppressor of cytokine signaling 1 (SOCS1) and SOCS3 showed higher degree values and interactions to each other. In another PPI network associated with neurological damage, as shown in Fig. 6B, a total of 45 nodes with 90 edges were observed, amongst which the bradykinin receptor B1 (BDKRB1), IL6, cholinergic receptor muscarinic 4 (CHRM4), luteinizing hormone/ choriogonadotropin receptor (LHCGR) and dopamine receptor D4 (DRD4) represented higher degree values. In addition, the PPI network related to gene transcription and translation is composed of 96 nodes and 1,128 edges (Fig. 6C). The top nodes with higher degrees in the PPI network include the nuclear cap binding protein subunit 1 (NCBP1), elongation factor tu GTP binding domain containing 2 (EFTUD2), ribosomal protein family (RPS3, RPS13, RPS8, RPSA, RPS12, RPS15 and RPS3A) and down-regulated eukaryotic translation initiation factor 3 family (EIF3A, EIF3B, EIF3E, EIF3H, EIF3I and EIF3J).

## Discussion

MD pathogenesis is very complex and the natural course of the disease following infection has been well established as the ‘Cornell Model’^4, 5^. Previous studies on host gene expression profiles in GaHV2-infected birds or cell lines have usually been performed using microarrays and/or qRT-PCR analysis^11, 12, 15, 20, 21^. Compared to qRT-PCR and microarrays, RNA-seq is a more powerful, species-specific and accurate technique and has been used as a high-throughput procedure for identifying novel and detailed molecular mechanisms in an increasing number of diseases^22–26^. This study has provided the observations on the transcriptional changes in the host’s response to GaHV2 infection and the progression of MD. Based on the data from RNA-seq analysis, large numbers of genes were found to show a change in their level of expression, and these genes were clustered analyzed, functionally annotated and further illuminated the significance of the changes.

When MD was first reported, it was primarily named as fowl paralysis (polyneuritis). According to the neurological clinical signs in MD the neurological syndrome was divided into four different types: classical transient paralysis, acute transient paralysis, persistent neurological disease and late paralysis^27^. When severe clinical neurological signs appeared, the expressions of iNOS, IFNG, ILB, IL6, and IL8 genes were elevated dramatically in the brain following the very virulent plus (vv+) GaHV2 infection, especially at 10 and 14 dpi^28^. For MD the neurological syndromes share similarities to the suspected autoimmune polyneuropathies such as CIDP (chronic inflammatory demyelinating polyneuropathy). Another important pathway related to the transmission of neural signal, ‘Neuroactive ligand-receptor reaction’, was persistently enriched from 14 to 60 dpi. The molecular events happening in the peripheral nerve system are consistent with the clinical neurological symptoms of GX0101-infected birds during the experimental time period from 7 to 90 days^29^. These data suggest that the neurological damage caused by very virulent (vv) GaHV2 infection appears early in viral infection and persist for the whole course of the disease. In BDKRB1-deficient mice, a reduced accumulation of polymorphonuclear leukocytes in inflamed tissue and significant analgesia to heat and capsaicin stimulation had been previously observed^30^. The BDKRB1 agonist markedly reduces clinical symptoms of experimental autoimmune encephalomyelitis in mice, whereas its antagonist leads to accelerated disease onset and greater severity of the disease^31^. In our study, the up-regulated BDKRB1 represents a node with a high connectivity amongst the genes involved in the PPI network associated to ‘neural signal’. Whether the increase of BDKRB1 expression could be influencing the neurological symptoms deserves to be further studied.

The JAK/STAT signaling pathway is involved in controlling multiple biological processes such as cell differentiation, proliferation, development, apoptosis and inflammation^32^. The negative regulators of JAK/STAT pathway include three major classes, SOCS family, PIAS family, and tyrosine phosphatases^33^. In our study, the JAK/STAT pathway was significantly enriched at 14 dpi, accompanying several up-regulated genes such as, IFNG, IL10, IL6, SOCS1, SOCS3, BclXL, PIAS1, PIAS2 and PIAS3. The expression of SOCS1, SOCS2, SOCS3 and CIS is at low levels when the body is in a steady state, but they will be rapidly increased by key cytokines^34^. In the presently constructed PPI network related to JAK/STAT and its negative regulators, the up-regulation of IFNG, IL6, IL10, SOCS1, SOCS3, and CIS was simultaneously observed. Previous studies have demonstrated that IL10 induces SOCS3 through the activation of STAT3, however, SOCS3 does not inhibit IL10 signaling, but rather counteracts the pro-inflammatory action of IL6, thus promoting the anti-inflammatory actions of IL10^35, 36^. The interaction between IL6, IL10, SOCS1, SOCS3, CISH and IFNG observed in the network is a compatible with the finding reported previously. In addition, BclXL was observed to be significant up-regulated in JAK/STAT signaling. The function of BclXL is anti-apoptotic^37^ and its up-regulation is possibly beneficial for GaHV2 by boosting the survival of host cells.

During the GaHV2 infection, induction of rapid-onset lymphomas and host death would not be beneficial for GaHV2 and thus a strategy of establishing a latent infection gives a longer persistence and increased reproduction. It has been suggested that the gross lymphomas might be an incidental consequence from increasing the state of activation, lifespan, and proliferative potential of latently infected lymphocytes^2^. In this phase, the cells are activated and undergo uninterrupted replication. This is well reflected in the molecular events elucidated in our present study where multiple significant pathways associated with gene transcription and translation, such as the ‘Ribosome’, ‘Ribosome biogenesis in eukaryotes’, ‘Spliceosome’, ‘RNA transport’ and ‘mRNA surveillance pathway’, were enriched during 3–4 weeks post GaHV2 infection. This is similar, but more extensive, to a previous report in which analogous pathways of ‘Protein transport’, ‘mRNA processing’ and ‘RNA splicing’ were significantly enriched at 21 and 28 dpi in thymus of GaHV2-infected birds^38^. In the process of protein synthesis, translation initiation is considered as a rate-limiting step and is governed by the availability and activity of EIFs^39^, amongst which EIF3 is the most complex member^40^. Depending on TC, EIF1, EIF1A and RNA oligonucleotides, mammalian EIF3 binds to the 40 S ribosomes in the absence of other EIFs and stimulates the assembly of the 43S pre-initiation complex^41^. In mRNA recruitment^42^, EIF3 enables a bridge to form the 40 S subunit and EIF4F-mRNA complex. Apart from the versatile role of EIF3 in translation initiation, the deregulations of EIF3 has been frequently observed in various human tumors^43^. Presently, the altered expression of EIF3 family was observed in multi-stages following GaHV2 infection. Further analysis of the PPI networks related to gene transcription and translation had demonstrated that the EIF family members, such as EIF3, EIF4 and EIF5, represent high degree values compared to the other genes. Their potential roles and underlined molecular mechanisms involved in MD biology need further investigations.

## Methods

### Ethics Statement

All experimental protocols were approved by the Laboratory Animal Management Committee of Key Laboratory of Animal Immunology, Ministry of Agriculture, China. Animal experiments with chickens were conducted following the protocols of the Laboratory Animal Management Committee of Key Laboratory of Animal Immunology, Ministry of Agriculture. China approved the permit (permit no. 2007001).

### Animal experiments

The animal experiments were conducted following the protocols of the Ethical and Animal Welfare Committee of Key Laboratory of Animal Immunology, Ministry of Agriculture, China, material from which has been reported by Yu *et al*.^29^ and Teng *et al*.^44^. Briefly, one-day-old white Leghorn SPF chickens (Jinan SPF Egg & Poultry Co. Ltd., China) were separately challenged with CEFs containing 2,000 PFU of GX0101 virus by abdominal cavity inoculation. Equal doses of mock CEFs serve as negative controls. At 3, 7, 14, 21, 30 and 60 days post-infection (dpi), three birds from each group were randomly selected and humanely euthanized. Spleens were collected and stored at −80 °C for subsequent study.

### cDNA library construction and RNA-seq

Total RNA was extracted from the spleens of GX0101-infected or mock-infected birds using TRIzol Reagent (Life Technologies, Carlsbad, CA, USA) following the manufacturer’s instructions. The concentration of the RNA was calculated by measuring the optical density at 260 nm (OD_260_) and OD_280_ spectrophotometrically (NanoDrop, Thermo Scientific, MA, USA). The RNA integrity number (RIN) was checked on Bioanalyzer 2100 (Agilent Technologies, Santa Clara, CA, USA). The RNA showing RIN values of ≥7.9 with the ratio of 28 S/18 S > 1.0 was used for further RNA-seq. For each time point, total RNA from the spleens of GX0101-infected or mock-infected birds were equally mixed for constructing cDNA libraries according to standard procedures. Briefly, mRNAs were purified from 10 μg of total RNA using oligo dT magnetic beads, followed by fragmenting the RNA into small pieces. The cleaved RNA fragments were used as templates to synthesize the first-strand and then the second-strand, and purified according to the instructions of QIAquick PCR Purification Kit (Qiagen). The eluted and purified double cDNAs were end-repaired, A-added, and adapter-ligated. The short fragments were then enriched by PCR amplification to construct libraries. Finally, sequencing was performed at the channels of an Illumina HiSeq^TM^ 2500 (Illumina, San Diego, CA, USA) with single end 50 bp. All raw data have been deposited in the NIH Short Read Archive database (SRP086669).

### Data processing

The Perl script was used to trim the original data (raw data) obtained from the sequencing system containing the reads of contaminated adapters, the low-quality reads, and the reads containing poly-Ns. The clean data were filtered statistically for the quality and data quantity, including Q30 statistics, data quantity statistics, base content statistics, etc. Reference gene and genome annotation files were downloaded from the UCSC (http://hgdownload.soe.ucsc.edu/goldenPath/galGal4) to build the reference genome library using ‘Bowtie2’ (v2.2.3), and then the clean data were mapped to the reference genome by ‘TopHat’ (v2.0.12). Quantification scores for all chicken genes and Reads per Kilobase Million Mapped Reads (RPKM) values were calculated using ‘Cufflinks’ (v2.0.2), which correct the transcript length and total numbers of mapped reads from the library to compensate different read depths for different samples. In addition, ‘HTSeq’ (v0.6.0) was run to calculate read counts for each gene. All data were analyzed using R Statistical Environment (http://www.r-project.org/), accompanied with an additional package of ‘gplots’.

### Differentially expressed gene analysis

The differentially expressed genes (DEGs) were analyzed using DESeq software by performing a pairwise comparison between each GX0101-infected and mock-infected libraries. The significance threshold of *p*-value in multiple tests was set by false discovery rate (FDR). The threshold, the absolute values of fold change ≥2 and FDR < 0.05, was applied to judge the significance of gene expression differences. Cluster analysis for all DEGs between GX0101-infected groups and mock controls was performed using a hierarchical cluster algorithm in R with a centroid method and Euclidean distance. For the functional and pathway enrichment analysis, the DEGs were then mapped into GO terms and the KEGG databases, significantly enriched GO and KEGG terms were determined by *p*-value ≤ 0.05.

### PPI network and module construction

The online software STRING (Search Tool for the Retrieval of Interacting Genes) (http://string-db.org/)^45^ was used for searching for the interactions of the proteins encoded by the DEGs. The combined score >0.4 was used as the cut-off criterion. Subsequently, the PPI networks were visualized using Cytoscape software (http://www.cytoscape.org)^46^ and the nodes with higher degrees of connectivity were taken as hub nodes.

### Quantitative real-time PCR analysis

Initially, a total of 12 genes from the DEGs were randomly selected for validating the results of RNA-seq using quantitative real-time PCR (qRT-PCR) analysis. For the confirmation of hub nodes in PPI networks, the other 21 genes were selected and similarly determined by qRT-PCR. The *GAPDH* gene was used as an internal reference. Gene-specific primers, as listed in Table S4, were designed and synthesized by Sangon Biotech Co., Ltd. (Shanghai, China). The RNA samples used for qRT-PCR assays were the same as used for RNA-seq experiments. The first strand cDNA was synthesized using the PrimeScript^TM^ RT reagent Kit with gDNA Eraser (TaKaRa) according to the manufacturer’s instructions. Genomic DNA was eliminated by treatment with gDNA Eraser, which has potent DNA degrading activity, at 42 °C for 2 min. All cDNA samples were diluted to 50 ng/μl before running qPCR reactions on an ABI PRISM 7500 Fast Real-Time PCR System (Applied Biosysterm, Foster City, CA, USA). The amplifications were performed in a total volume of 20 μl reaction mixtures included 10 μl of 2 × SYBR^®^ Premix Ex Taq (Tli RNaseH Plus) reagent, 100 ng of cDNA and 0.4 μl of each primer (10 μM). The thermal cycling profile consisted of an initial denaturation at 94 °C for 3 min, followed by 40 cycles of denaturation at 95 °C for 15 s and annealing/extension at 60 °C for 40 s. An additional temperature-ramping step from 60 °C to 95 °C was used to produce the melting curve. All reactions were conducted in triplicate and included negative controls without templates. Expression differences between GX0101-infected and mock-infected birds were calculated using 2^−ΔΔCt^ method. The expression levels of target genes were normalized to the transcription levels of the *GAPDH* gene in the same samples.

## Electronic supplementary material

Supplementary information

Dataset 1

Dataset 2

Dataset 3

Dataset 4
