# Supplementary material for: Dynamic Changes in the Splenic Transcriptome of Chickens during the Early Infection and Progress of Marek’s Disease
Source: Sci Rep. 2017 Sep 14;7:11648. doi: 10.1038/s41598-017-11304-y (PMC5599560; doi:10.1038/s41598-017-11304-y)
Supplement: Supplementary file 1 — Supplementary information [file 41598_2017_11304_MOESM1_ESM.doc]

**Title:**

**Dynamic Changes in the Splenic Transcriptome of Chickens during the Early Infection and Progress of Marek’s Disease**

**Authors**:

Lu Dang^1,2,#^, Man Teng^2,#^, Hua-Wei Li^1,2^, Hui-Zhen Li^2,3^, Sheng-Ming Ma^2,4^, Pu Zhao^2^, Xiu-Jie Li^2^, Rui-Guang Deng^2^, Gai-Ping Zhang^1,3,5,*^, Jun Luo^2,4,*^

**Affiliation**:

^1^ College of Veterinary Medicine, Northwest A&F University, Yangling 712100, People’s Republic of China;

^2^ Key Laboratory of Animal Immunology of the Ministry of Agriculture, Henan Provincial Key Laboratory of Animal Immunology, Henan Academy of Agricultural Sciences, Zhengzhou 450002, People’s Republic of China;

^3^ College of Animal Science and Veterinary Medicine, Henan Agricultural University, Zhengzhou 450002, People’s Republic of China;

^4^ College of Animal Science and Technology, Henan University of Science and Technology, Luoyang 471003, People’s Republic of China;

^5^ Jiangsu Co-innovation Center for Prevention and Control of Important Animal Infectious Diseases and Zoonoses, Yangzhou 225009, People’s Republic of China.

^#^ These authors contributed equally to this work.

**^*^ Corresponding authors:**

***Jun Luo***, E-mail: [luojun593@aliyun.com](mailto:luojun593@aliyun.com)

Mailing address: Key Laboratory of Animal Immunology, Henan Academy of Agricultural Sciences, No.116 Huayuan Road, Zhengzhou 450002, People’s Republic of China

Phone: +86-371-65756056

Fax: +86-371-65738179

***Gai-Ping Zhang***, E-mail: [zhanggaiping2003@163.com](mailto:zhanggaiping2003@163.com)

Mailing address: College of Animal Science and Veterinary Medicine, Henan Agricultural University, No.63 Nongye Road, Zhengzhou 450002, People’s Republic of China

Phone: +86-371-63550369

Fax: +86-371-63558998

**Figure S1**

**
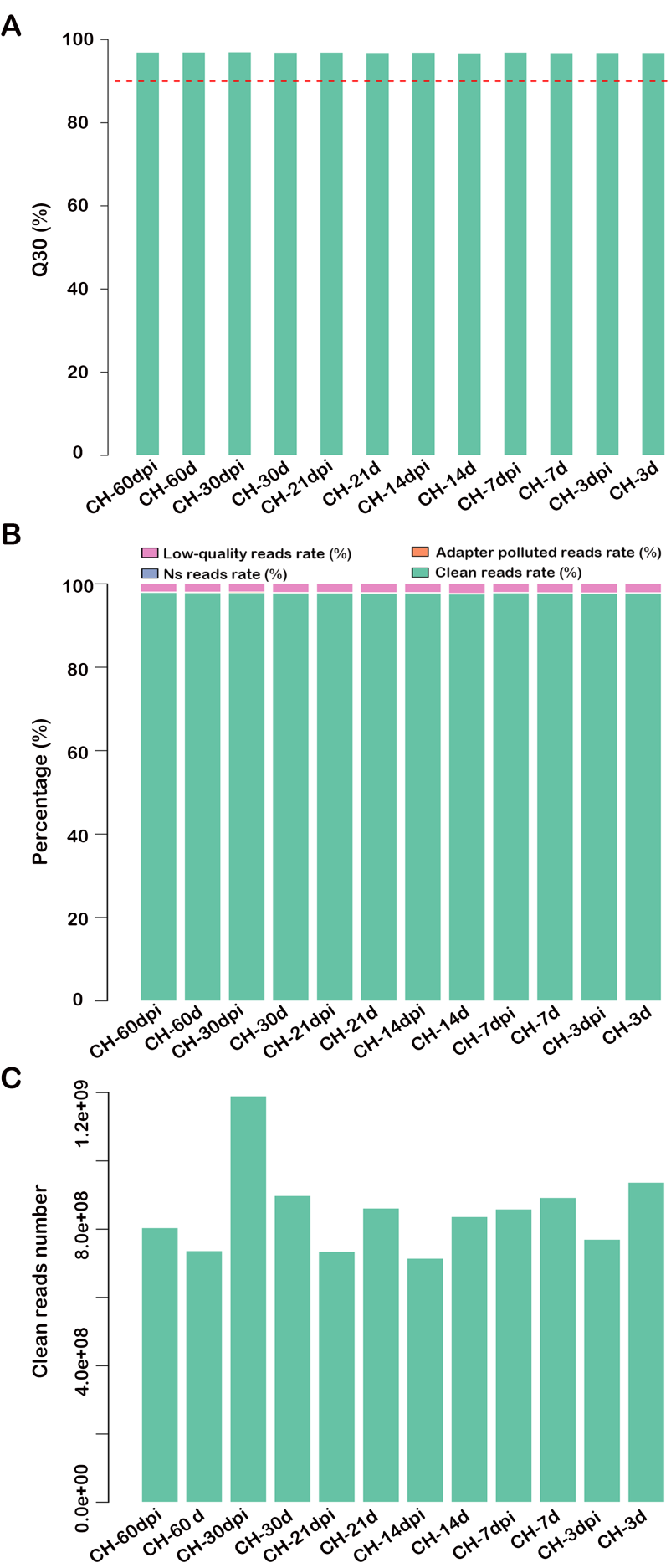
**

**Fig. S1.** Overview of RNA-seq data of twelve cDNA libraries. (A) The quality control graph of Q30 (false discovery rate ≤ 0.1%); (B) Filter distribution; (C) The quantitative distribution of clean reads.

**Figure S2**

**
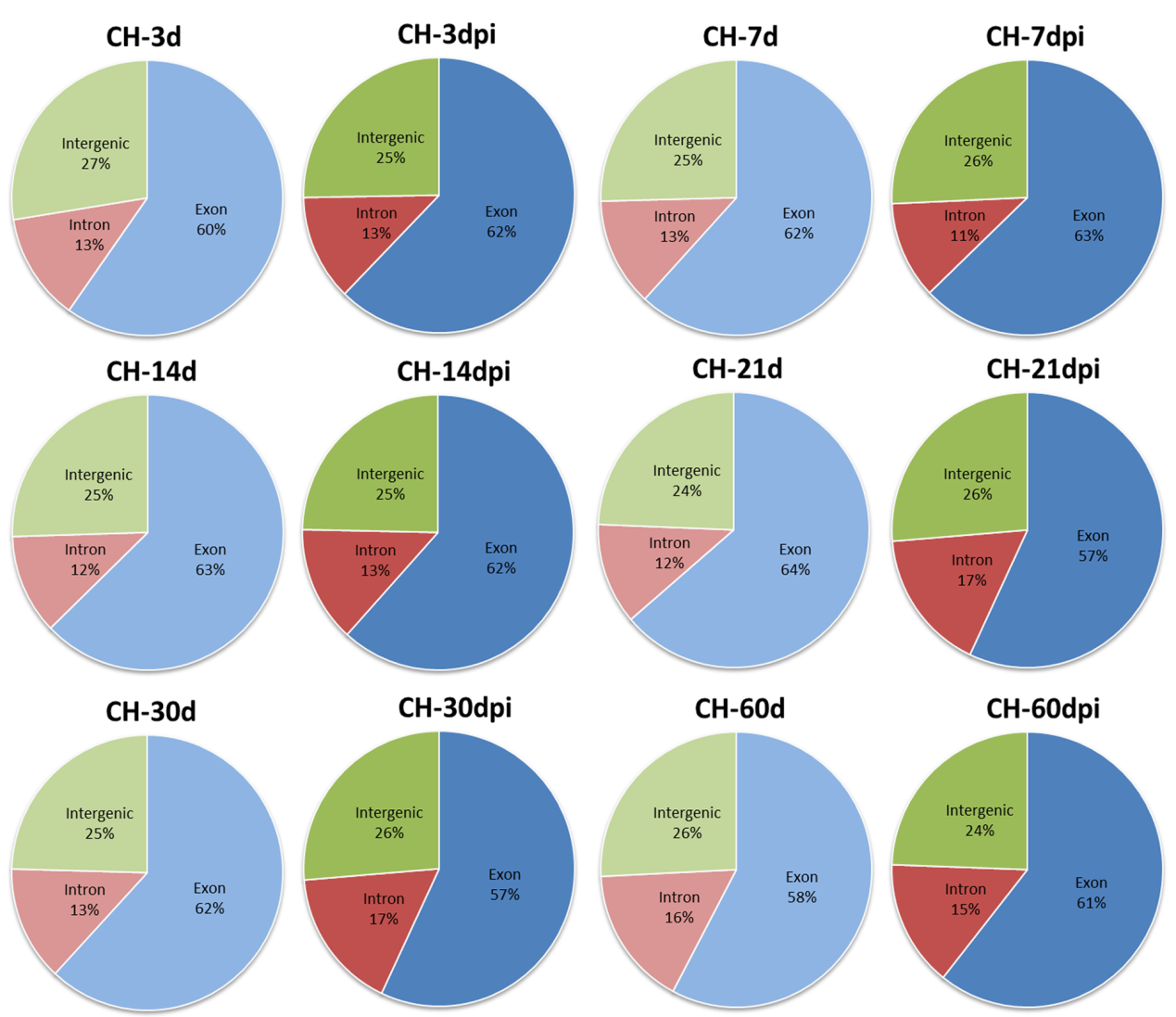
**

**Fig. S2.** Distribution diagrams of unique mapped reads aligning to chicken genome. For each time point, the cDNA libraries constructed for GX0101-infected birds or mock controls were named as CH-#dpi and CH-#d, respectively.
